# Organo/Zn-Al LDH Nanocomposites for Cationic Dye Removal from Aqueous Media

**DOI:** 10.1186/s11671-016-1402-0

**Published:** 2016-04-27

**Authors:** G. Starukh, O. Rozovik, O. Oranska

**Affiliations:** Chuiko Institute of Surface Chemistry of the National Academy of Sciences of Ukraine, 17 General Naumov Str., Kyiv, 03164 Ukraine

**Keywords:** Layered double hydroxides, Organo/LDH nanocomposites, Intercalated surfactants, Cationic dye sorption, Wastewater treatment

## Abstract

Cationic dye sorption by Zn-Al-layered double hydroxides (LDHs) modified with anionic surfactants was examined using methylene blue (MB) dye as a compound model in aqueous solutions. The modification of Zn-Al LDHs was performed by reconstruction method using dodecyl sulfate anion (DS) solutions. DS contained Zn-Al LDHs were characterized by XRD, FTIR, thermogravimetric, and SEM analysis. The reconstructed organo/Zn-Al LDHs comprise the crystalline phases (DS-intercalated LDHs, hydrotalcite), and the amorphous phase. The intercalation of DS ions into the interlayer galleries and DS adsorption on the surface of the LDHs occurred causing the MB adsorption on the external and its sorption in the internal surfaces of modified LDHs. The presence of DS greatly increased the affinity of organo/Zn-Al LDHs for MB due to hydrophobic interactions between the surfactants and the dye molecules. The optical properties of sorbed MB were studied.

## Background

Facing the ever-growing demand for materials combining several properties, research is now extensively devoted to the preparation of multifunctional assembly in which the different components may act in synergism. In such approach, the layered compounds are of great interest [1], and some interesting multifunctionalities are reported such as drug delivery systems using lamellar nanocomposites with incorporated drug [2], the flexible solar cell using layered compound-metal particle composites [3], and flat-panel displays using graphene oxide liquid crystals [4].

A special attention is now paid to the layered double hydroxides (LDHs) because of their tunable charge density and large chemical versatility [5, 6]. LDHs or hydrotalcite-like compounds are a class of ionic lamellar compounds made up of positively charged brucite-like layers with an interlayer region containing charge compensating anions and solvation molecules. The metal cations occupy the centers of edge-sharing octahedra, whose vertexes contain hydroxide ions and that are connected to form infinite 2D sheets. The chemical composition of LDHs is expressed by the general formula [M^2+^_1−x_M^3+^_x_(OH)_2_][A^n−^]_x/n_·zH_2_O, where M^2+^ may be common; Mg^2+^, Zn^2+^, or Ni^2+^, and M^3+^ may be common; Al^3+^, Ga^3+^, Fe^3+^, or Mn^3+^. A^n−^ is nonframework charge-compensating inorganic or organic anion, e.g., CO_3_^2−^, Cl^−^, SO_4_^2−^, and RCO^2−^, and *x* is normally between 0.2 and 0.4. LDHs layers gain a positive charge by isomorphous substitution of M^3+^ for M^2+^, which is compensated by interlayer anions [5].

Some attempts have been made to prepare the intercalated compounds with a large variety of anionic species for various purposes and applications [7–9]. Recently, LDHs began to be used in the removal of inorganic and organic contaminants. Grover et al. [10] used synthetic Mg-Al LDHs to the uptake of arsenite ions from aqueous solution by an ion-exchanged process. Alves and coworkers [11] reported that Mg-Al LDHs removed the colored oxidation debris from wastes originated from carbon nanotubes chemical processing. Calcined LDHs were effectively used to the uptake of anionic dyes from wastewater by sorption in the interlayers, and/or adsorption on the external surfaces of LDHs [12, 13]. As the inorganic LDHs host lamella improve the thermal and photochemical stability of the dyes [14], the dye sludge can be reutilized as colorant filler for polymer materials exhibiting the resistance to bleeding and fire. The LDH-based pigments can be applied for the production of coatings, paints, and composite materials [9].

Due to their anionic exchange capacity, LDHs are suitable for intercalation and sorption of negatively charged species but are not applicable for positively charged ones. Recently, some researchers used anionic surfactants to modify LDH’s surface properties, and then to adsorb many types of organic molecules [15–17]. The organic phase in the interlayer which was formed with the intercalated organic ions, acted as an adsolubilization medium increasing the affinity of LDHs for organic compounds. Sodium dodecyl sulfate-modified LDHs demonstrated the greatly enhanced sorption of ionizable and nonionic organic contaminants such as pesticides, herbicides, and aromatic hydrocarbons [16, 18, 19]. Surfactant-intercalated LDHs allowed obtaining an organic–inorganic hybrid pigments based on cationic dyes [20]. Mg-Al LDHs intercalated with DS were effectively used for the removal of cationic dyes as safranin [21] and methylene blue [22].

There are several ways of LDHs modification reported in literature, for example, co-precipitation in the presence of organic species, ion exchange method, reconstruction method, etc. [5, 6]. Among these, the reconstruction method has been found to be more facile and could be applied to carry out the modification of large amounts of LDHs at a time. This method is based on a unique property of the layered materials. Most of the LDHs clays can regenerate an original structure from their oxide form, when the latter is dispersed in an aqueous solution containing the anion present in the original material. This phenomenon was also confirmed for obtaining of DS-modified Mg-Al LDHs [17].

Sodium dodecyl sulfate is a widespread contaminant of aquatic environmental as it is intensively used in many industrial processes, such as colloids stabilization, metal treatments, mineral flotation, and production of daily-used detergents and pesticides [23, 24]. Adsorption technology can offer a potential low-cost treatment of this compound, by using soil, activated carbon, and clays, but after DS adsorbing, their products were difficult to recycle [23–25]. It follows that the LDHs are reasonable to use for DS removal and to exploit the obtained product as valuable resources to dispose organic matter from wastewater.

The investigation of Zn-Al interlayers modified with DS using reconstruction method is scarcely reported in the literature. The published results were devoted to DS-intercalated Zn-Al LDHs synthesized by direct co-precipitation and ion-exchange methods. Therefore, the purpose of this study was to synthesize the surfactant-modified LDHs by reconstruction of calcined Zn-Al LDHs in solutions with different DS concentration and to evaluate the sorption performance of the prepared materials as for the capacity of cationic dye (methylene blue) removal from water, comparing with the sorption capacity of unmodified Zn-Al LDHs.

## Methods

### Preparation of Zn-Al LDHs

All chemicals were analytical grade and used without further purification. Zn-Al LDHs with carbonate as the interlayer anion, with [Zn]/[Al] = 1/2 were synthesized by co-precipitation at a constant pH 10, following the method described in [5]. A mixed solution of 0.1 mol of Zn(NO_3_)_2_ and 0.05 mol of Al(NO_3_)_3_ in 200 ml of distilled water was added dropwise under vigorous stirring to 200 ml of an aqueous solution containing 0.3 mol of NaOH and 0.1 mol of Na_2_CO_3_. The pH 10 was maintained constant by the addition of NaOH. Once addition was completed, the temperature was raised up to 85 °C and the slurry was being kept for 6 h at this temperature under continuous stirring. After that, the slurry was cooled down to the room temperature within several hours. The product was isolated by filtration and washed several times with the deionized water until pH 7. Afterwards, the solid was dried at 100 °C. The sample was labeled as ZnAl LDH.

The Zn-Al LDHs were modified with sodium dodecyl sulfate (CH_3_(CH_2_)_11_SO_4_Na) by reconstruction method. Zn-Al LDHs have been calcined at 450 °C over 2 h to destroy the layered structure. The suspensions contained 1 g of calcined LDHs and 50 ml of CO_2_ —free aqueous solutions of DS were stirred for 24 h at room temperature. The concentration of DS ranged from 0.012 mol L^−1^ to 0.205 mol L^−1^. This sample was labeled as ZnAl LDH/DS.

### Characterization

XRD patterns of the samples were recorded with the DRON-4-07 diffractometer (CuK_α_ radiation). Calculation of apparent crystallite size for LDHs has been performed by Debye-Scherrer formula: *β*(2*θ*) = 0.94*λ*/(*D*cos *θ*°), using (003) and (110) reflection, employing the FWHM procedure. The thermogravimetric analysis (TGA) and differential thermal analysis (DTA) were carried out using Derivatograph Q-1500 D MOM (Hungary) equipment operated under a flow of an air at the heating rate of 10° min^−1^. Infrared spectra were obtained in the range of 4000–400 cm^−1^ on a Thermo Nicolet NEXUS FT-IR spectrophotometer (Nicollet, USA). The morphology and microstructure of Zn-Al LDHs were examined by the scanning electron microscope (SEM; JSM-6490-LV, JEOL, Japan). Diffuse reflectance spectra were obtained with a Lambda 35 UV–Vis (Perkin Elmer) spectrometer equipped integrated with Labsphere RSA-PR-20 in the range of wavelength 200–1000 nm. The UV–visible spectra of the solutions were recorded on a Lambda 35 UV–Vis (Perkin Elmer) spectrometer using a quartz cell (1-cm path length), with distilled water as a blank.

### Batch Adsorption

MB adsorption isotherms were determined using a batch adsorption approach. Typically, 0.02 g of freshly as-synthesized and calcined Zn-Al LDHs were introduced into 100-mL glass tubes containing 40 mL of freshly prepared aqueous MB solutions with concentrations ranging from 8 × 10^−7^ mol L^–1^ to 5 × 10^−5^ mol L^−1^. The adsorption process was allowed to last 4 h under continuous stirring. The particles were removed by centrifugation at 6000 rpm, and the residual concentration of MB in the solution was determined using UV–Vis spectrometry at a detecting wavelength of 663 nm. The equilibrium adsorption amount of MB in the sample was calculated according to equation: *q*_*е*_ = (*С*_0_ − *С*_*е*_)*V*/*m*, where *q*_*e*_ is the amount of MB adsorbed at equilibrium, *C*_0_ is the initial MB concentration, *C*_*e*_ is the equilibrium concentration in solution, *V* is the total volume of solution, and *m* is the sorbent mass.

## Results and Discussions

### Characterization

**Fig. 1:**
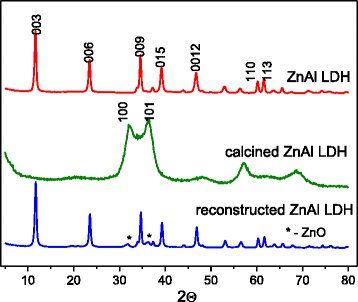
X-ray diffraction patterns of as-synthesized, calcined, and reconstructed ZnAl LDH

The XRD pattern of the as-synthesized ZnAl LDH contains the characteristic reflections of layered double hydroxides with the basal planes of (003), (006), and (009) peaks at low 2*θ* angles and the other peaks for (101), (015), (0012), (110), and (113) planes at high 2*θ* angles (Fig. 1). The unit cell parameters and the crystallite size for the ZnAl LDH were calculated, and the results are listed in Table 1. The *c* parameter corresponds to three times the distance between adjacent brucite-like layers, and the *a* parameter is almost the same as that of brucite for which *a* = 3.10 Å. The basal spacing corresponds to CO_3_^2−^-containing LDHs. These values are in the literature range [26]. All the reflections are sharp indicative of a highly crystalline material.

**Table 1:** Structural parameters of ZnAl LDHs

Sample	*a* = 2d_110_, Å	*c* = 3d_003_, Å	Basal spacing d_003_, Å	Interlayer distance^a^, Å	Crystal size, Å	
					d_003_	d_110_
ZnAl LDH	3.074	22.8	7.60	2.8	172	198
ZnAl LDH reconstructed	3.073	22.7	7.55	2.8	174	122
ZnAl LDH/DS	3.068	78.6	26.20	21.4	152	162

^a^Interlayer distance = d_003_—thickness of the brucite layer (4.8 Ǻ) [44]

ZnAl LDH was calcined in air at 450 °C for 2 h to obtain a mixture of zinc and aluminum oxides. The reflections in XRD pattern of calcined ZnAl LDH are characteristic of the ZnO (Fig. 1). It is known that at certain temperature, the original hydrotalcite is converted into a mixture of oxides which have “memory” of the original structure [26]. In fact, in the presence of aqueous solutions, the oxides regenerate the double hydroxides in the form of brucite-like sheets and the positive charges are balanced by anions presented in solutions. The hydration of calcined ZnAl LDH in aqueous suspension caused to the reconstruction of the hydrotalcite phase (Fig. 1). The source of anions in this case is atmospheric CO_2_ from which CO_3_^2−^ ions are formed.

**Fig. 2:**
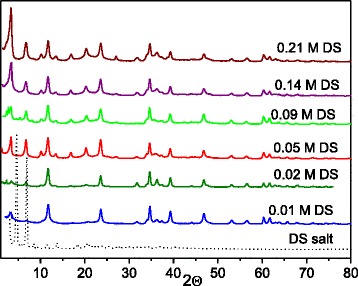
X-ray diffraction patterns of organo/Zn-Al LDHs obtained by reconstruction of calcined ZnAl LDH in DS solutions of various concentrations

The layered structure reconstruction of calcined ZnAl LDH was performed in aqueous solutions of different DS concentrations that corresponded to Al (ZnAl LDH):DS molar ratio in the range of 0–1.5. The appearance of new diffraction peak signed the formation of a new phase was observed at small angles indicating DS anion incorporation into the interlayer space (Fig. 2). The intercalated amount of DS ions was gradually accumulated with increase of its concentration in solutions used for modification of Zn-Al LDHs. The ratio of integral intensities of reflections that correspond to the intercalated (2*θ* = 3.4 °) and hydrotalcite (2*θ* = 11.7) phases was calculated (Table 2). The main type of intercalated derivatives was obtained having the mean interlayer spacing 26 Å, 2*θ* = 3.4 ° (Table 2). These interlayer distances depend upon the orientation of the chains within the interlamellar space [27]. As the length of sodium dodecyl sulfate molecule is 20.8 Å [28], the basal spacing of 26.8 Å corresponds to a perpendicular monolayer arrangement of DS ions between the host layers [29]. The products with basal spacing in the neighborhood of 36 Å could contain the DS bilayers which are partially overlapped [27]. The characteristic reflections of DS are absent in the XRD pattern of the organo/Zn-Al LDHs. The diffraction peaks at 2*θ* equal to 10.2°, 13.5°, 16.9°, and 20.3° are suggested to correspond with the formation of a superlattice consisted probably of ordered DS ions in the interlayer spaces (Fig. 2).

**Table 2:** Al (ZnAl LDH):DS molar ratio, DS concentration, d-spacing of LDH/DS, and hydrotalcite phase content of ZnAl LDH modified with DS

Al(ZnAl LDH):DS molar ratio	C_DS_, mol L^−1^	Basal spacing d_003_, Å	Integral intensities I (3.4°)/I (11.7°) ratio	Hydrotalcite phase content, %
1:0.08	0.01	7.6; 26.4; 38.4	0.33	16
1:0.16	0.02	7.6; 26.4	0.39	26
1:0.33	0.05	7.6; 26.4	0.82	21
1:0.65	0.09	7.6; 26.4	0.95	10
1:1.00	0.14	7.6; 26.4	2.33	18
1:1.50	0.21	7.6; 26.4	3.76	12

To evaluate the content of hydrotalcite phase in DS-modified Zn-Al LDHs, the quantitative phase analysis was performed using the additives method [30]. We assumed an insignificant change in the mass absorption coefficient of the ZnAl LDH modified with DS after the addition of ZnAl LDH reference. In this case, the peak intensity of hydrotalcite in the first approximation is a linear function of the additive concentration. The content of hydrotalcite in the samples was graphically determined. The obtained data have to be treated as estimated (the accuracy of the method is 90 %), as the equality of mass absorption coefficients of examined sample and additive sample was assumed (Table 3). It has to be pointed that the content of the hydrotalcite phase is in the range of 10–26 %. This can be an evidence of the imperfection of crystalline structure of ZnAl LDH modified with DS. According to [31], the extra-phases coexist in the LDHs. The as-synthesized and the reconstructed Zn-Al LDHs contained approximately 25 and 23 wt.% of an amorphous phase [31]. The authors reported that the reconstructed samples contained an additional about 3 wt.% of ZnO phase (zincite) appeared from the segregation of Zn from the brucite-like layers. It seems that the modification of ZnAl LDHs with DS causes to the additional formation of the amorphous phase.

**Table 3:** The thermal decomposition stages of Zn-Al LDHs

Samples	Temperature intervals, °C				Total mass loss, %
	60–190	190–300	300–500	500–950	
ZnAl LDH	4.2	20.9	4.4	2.6	32.1
ZnAl LDH reconstructed	3.9	17.6	4.0	2.0	27.5
ZnAl LDH/DS (Al:DS = 1:0.33)	6.7	28.3	7.6	8.8	51.4

**Fig. 3:**
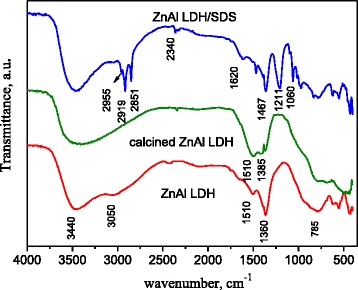
FT-IR spectra of as-synthesized, calcined, and DS-modified ZnAl LDH

The presence of anionic surfactants in the LDH structure can be further supported by FT-IR spectroscopy. The IR spectra of the as-synthesized ZnAl LDH, its calcined forms, and DS-modified ZnAl LDH are presented at Fig. 3.

LDHs containing CO_3_^2−^ anions have the characteristic bands for various modes of infrared sensitive vibrations of the anion. In most LDHs, these bands are observed in the range of 850–880 cm^−1^ (bending non-planar mode), 1350–1380 cm^−1^ (the asymmetric stretching mode), and 670–690 cm^−1^ (the bending angular mode) [5]. In the case of the Zn-Al LDHs, the sharp band at 1360 cm^−1^ without any distinct shoulder or degeneration indicating the absence of splitting of the asymmetric stretching vibrations (Fig. 3). It has been explained [32] by the fact that carbonate ions occupy a highly symmetric site between the hydroxide sheets. A small shoulder around 690 cm^−1^ in ZnAl LDH spectra indicates the bending angular mode vibrations and the band around 870 cm^−1^ indicates that the bending non-planar vibrations are overlapped with stretching M–OH vibrations (Fig. 3). A broad band at 3200–3700 cm^−1^ is observed for all the compounds. It is attributed to OH stretching vibrations of the octahedral layer and intercalated water molecules [33]. The shoulder at 3050 cm^−1^ of IR spectra of ZnAl LDH is attributed to hydrogen bonding of H_2_O molecules to CO_3_^2−^ ions in the interlayer space [34]. The bands at 1510 and 1385 cm^−1^ are signed to polydentate carbonates while the sharp band at 2340 cm^−1^ corresponds to the vibrations of gaseous CO_2_ present in the experimental conditions. The vibrations below 700 cm^−1^ are characteristic to metal-oxygen bond stretching. The various lattice vibrations associated with metal hydroxide sheets caused to the appearance of the sharp bands around 780, 550, and 430–450 cm^−1^ [17].

In the case of calcined LDHs, the absorption at 3800–3500 cm^−l^ is pointed to the stretching vibrations of surface-free hydroxyl groups. The bands at 1510 and 1385 cm^−1^ are due to residual bulk polydentate carbonates while the sharp bands at 2340 cm^−1^ belong to gaseous CO_2_ present in the experimental conditions. Thus, the crystal-layered structure is destroyed after calcination of LDHs at 450 °C (as was confirmed with the XRD pattern in Fig. 1), but the calcined products still contain the interlayer carbonate anions and bounded water.

IR spectrum of the ZnAl LDH/DS contains the vibration of carbonate ions located at 1360 cm^−1^ that indicates the presence of carbonate form of ZnAl LDH. All the typical bands of DS were observed at corresponding wavenumbers, such as C–H stretching and bending bands (2840–2955 cm^−1^), –OSO_3_^−^ anions stretching and bending bands (1211, 1060, and 720 cm^−1^) [21]. The stretching vibrations of lattice water and –OH groups were appeared at 3480 and 3636 cm^−1^ as the strong overlapping bands. The peak at 1620 cm^−1^ was attributed to the H–O–H bending vibration of the interlayer water molecules in organo/Zn-Al LDH.

**Fig. 4:**
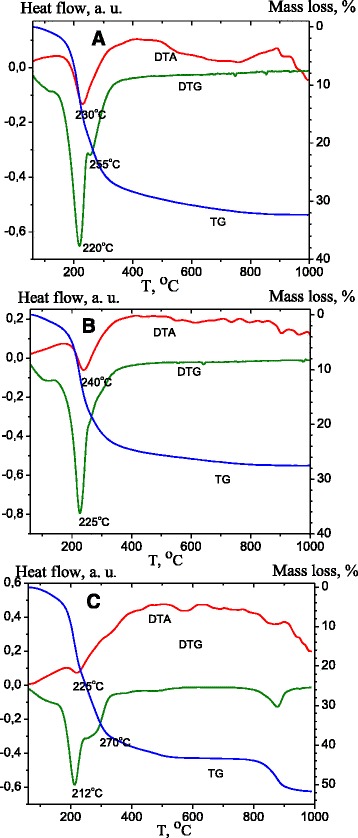
TG, DTA, and DTG curves as-synthesized (**a**), reconstructed (**b**), DS-modified (**c**) ZnAl LDH

The thermal decomposition of hydrotalcite-like compounds was studied. Figure 4 displays the TG − DTG − DTA curves of the as-synthesized, reconstructed, and DS-modified Zn-Al LDHs. The three events have been assigned to the thermal decomposition of LDH-carbonate: in the first one (60–190 °C), the mass loss is attributed to the removal of water from internal gallery surfaces and the external non-gallery surface; in the second interval (190–300 °C), the loss is ascribed to the dehydroxylation of the brucite-like sheets and removal of interlayer anions; in the last one (300–500 °C), the mass loss is recognized as the total dehydroxylation and collapse of the structure due to the removal of the remaining interlayer anions [35]. In order to study the contribution of water and CO_2_, mass losses were divided into three intervals: 60–190, 190–300, and 300–500 °C (Table 3). The small mass loss observed in the interval 500–1000 °C can be ascribed to the loss of some carbonate anions strongly adsorbed on the mixed oxides crystallites [36].

The smaller total mass loss of the reconstructed ZnAl LDH is explained by the fact that some part of calcined LDHs was not restored by hydration. So, the reconstructed LDH contained fewer hydroxides and carbonate ions in comparison with the as-synthesized ZnAl LDH. As shown by XRD analysis, the traces of ZnO are remained in the pattern of the reconstructed ZnAl LDH (Fig. 3).

The thermal decomposition of DS-modified LDH is almost identical to ZnAl LDH (Fig. 4c). The temperature of interlayer water loss seems to be lower than that of the as-synthesized and the reconstructed ZnAl LDH indicating the changes of LDH structure. It can be caused by the fact that DS ions intercalated into interlayer of LDHs reduce the interactions of water with the environment in ZnAl LDH. In other words, DS intercalation into ZnAl LDH increases the hydrophobic nature of LDH interlayer surface [16]. The second step of dehydroxylation of the brucite-like sheets is accompanied by DS ions destruction. The decomposition of DS ions takes place in the range 210–250 °C [37], and therefore, a greater loss is observed above 200 °C. The mass loss of DS-modified LDHs at 300–500 °C occurs due to the total dehydroxylation and the collapse of the layered structure. The thermogravimetric analysis coupled with mass spectroscopy revealed that the pyrolysis of the organic anion takes place under thermal treatment of LDHs [27]. The mass loss between 800 and 900 °C can be ascribed to SO_3_ evolution as a result of the decomposition of (Zn, Al) sulfate formed by decomposition of DS anions during the second mass loss stage [38]. The total mass loss of ZnAl LDH/DS is calculated as 51 % that is on 24 % more than the total mass loss for ZnAl LDH reconstructed in aqueous suspensions. This increase in the mass loss is resulted from the loading of DS, whose decomposition is reflected by the mass loss in 400–900 °C and the exothermal peak at 642 °C.

**Fig. 5:**
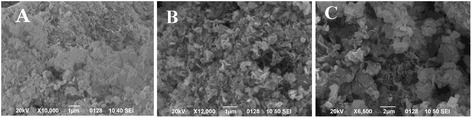
SEM images of ZnAl LDH (**a**), calcined ZnAl LDH (**b**), ZnAl LDH/DS (**c**)

The SEM images of the samples studied are presented in Fig. 5. The as-synthesized ZnAl LDH exhibited the characteristic hexagonal plate-like habit of anionic clays (Fig. 5a). The basal plane of the platelets is ranging from 200 to 500 nm. The calcination process does not alter the morphology and the size of the particles. The calcined ZnAl LDH sample retains platelet shape (Fig. 5b). It is known that mixed oxides have the ability to form paracrystalline phases that represent an intermediate phase between the crystalline and amorphous one [39]. Thermal decomposition of the LDHs leads to the formation of the metastable phase that contains divalent and trivalent cations with densely packed configuration [5]. The image of ZnAl LDH/DS demonstrates the aggregates of irregular flaky particles revealing that the modification with DS anions results in the aggregation of LDH crystals (Fig. 5c). A diameter of the basal plane DS-modified ZnAl LDH is higher (500–1100 nm) in comparison with the as-synthesized ZnAl LDH.

### Study of MB Dye Removal

The sorption properties of ZnA LDH modified with solutions of different DS concentration were investigated in removal of MB from 2 × 10^−4^ M solution (Table 4). The amount of sorbed MB is increased with increasing of DS quantity used for modification of Zn-Al LDHs. The organo/Zn-Al LDHs showed the high capacity to MB sorption while the as-synthesized and the calcined ZnAl LDH demonstrated very pure MB sorption (Table 4). The low MB adsorption on inorganic LDHs was also observed for Mg-Al LDHs [40]. ZnAl LDH in carbonate form was modified in 0.05 M DS solution and used for MB sorption. The sorption capacity of hydrotalcite with DS-modified external surface has been increased in comparison with the unmodified as-synthesized and the calcined ZnAl LDH, but it was still less than the sorption capacity of the organo/Zn-Al LDHs with intercalated DS ions (Table 4). While DS-modified ZnAl LDH with intercalated DS ions removed 75 % of MB from solution, the DS-modified hydrotalcite eliminated only 33 % of MB at the same conditions. So, the most of MB is sorbed on internal surface of LDH.

**Table 4:** Comparison of the MB sorption over ZnA LDH modified with DS solutions of various concentrations

Al (ZnAl LDH):DS molar ratio	C_DS_, mol L^−1^	q (MB), mmol g^−1^	q (MB), mg g^−1^	% of MB removal
1:0.08	0.01	0.07	22	19
1:0.16	0.02	0.28	90	70
1:0.33	0.05	0.30	96	75
1:0.65	0.09	0.29	93	73
1:1.00	0.14	0.31	99	78
1:1.50	0.21	0.35	113	89
ZnAl LDH	0	0.02	6	5
calcined ZnAl LDH	0	0.01	3	2
ZnAl LDH with adsorbed DS	0.05	0.13	42	33

[MB] = 2 × 10^−4^ mol L^−1^, [sorbent] = 0.5 g L^−1^

**Fig. 6:**
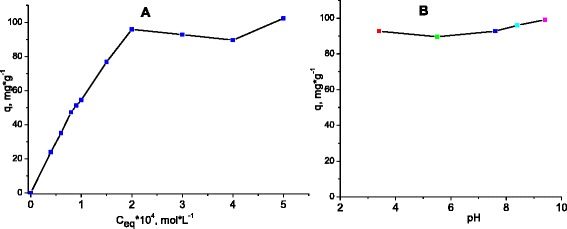
Isotherm of MB sorption by ZnAl LDH/DS (**a**); effect of pH on MB sorption by ZnAl LDH/DS (**b**)

The MB sorption isotherm for ZnAl LDH modified by the reconstruction of calcined ZnAl LDH in 0.05 M DS solution is presented in Fig. 6a. The amount of MB sorbed sharply increased at low MB concentration and then reached a plateau at the concentration higher than 2 × 10^−4^ mol L^−1^.

The results indicate that the organic phase (DS) greatly increased the affinity of organo/Zn-Al LDHs for MB due to hydrophobic and/or electrostatic interactions between the surfactants and the dye molecules. The pH value of the solution is an important parameter for control of the sorption process since it can affect sorption of the dye by changing the surface charge of the sorbents and ionization behavior of the sorbents and dye. The effect of pH on the removal of MB was studied using the dye concentration of 2 × 10^−4^ mol L^−1^ and at pH values in the range of 3–9. From the results presented in Fig. 6b, it was clear that the sorption capacity was not particularly dependent on pH. This might be due to the buffering properties of the DS intercalated sample.

The electrostatic attraction between the dye anions and the positively charged hydroxide surface in LDHs was the rate-limiting step in most of the surface adsorption studies. But in our case, the organic modification of ZnAl LDH produced high hydrophobicity due to the linear arrangement arising from the assemblage of DS anions. Such aggregation has been reported to enhance the adsorption capacity over a broad spectrum of dye molecules irrespective of their ionic charges and structure [41]. Under such circumstances, the hydrophobic/hydrophobic interactions between the alkyl chains of the surfactant and MB molecules are predominate over the electrostatic attractions. There was confirmed the prevailing influence of the hydrophobicity on the high adsorption of the cationic dyes by various sorbents modified with surfactants [18, 22].

**Fig. 7:**
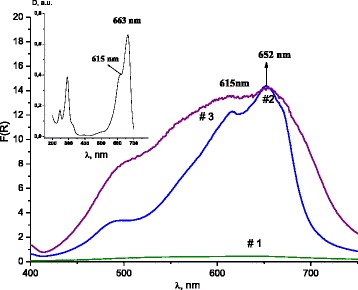
UV–Vis-DR spectra of ZnAl LDH (*#1*) and DS-modified ZnAl LDH after MB sorption from 10^−3^ M MB solutions (*#1*, *2*) and 5 × 10^−3^ M MB solutions (*#3*). Inset: UV–vis spectra of 10^−5^ M MB aqueous solution

The optical absorption of MB sorbed by ZnAl LDH/DS was studied. For dye molecules embedded into a host structure for any shift on the optical response suggests the occurrence of some changes in the electron density in the locality of the chromophore molecule which may be originated from an intermolecular interaction and/or from the guest-host accommodation [40]. The optical spectrum of initial MB aqueous solution contains in long-wavelength region of two absorption peaks at 615 and 663 nm corresponding to the dimmers and monomers, respectively (Fig. 7, inset). The shift in the absorption maximum of the monomer to a shorter wavelength is observed for MB sorbed on ZnAl LDH/DS (Fig. 7).

MB adsorbed on interlamellar clay sites can be distinguished spectroscopically from MB adsorbed on external surface sites [42]. The diffuse reflectance spectra of ZnAl LDH/DS with sorbed MB (*q* = 32 mg g^−1^) were analyzed (Fig. 7, #2). According to [43], the absorption band at 652 nm indicates the adsorption of MB monomers on the internal surface sites of organo/LDHs (Fig. 7). The diffuse reflectance spectra of ZnAl LDH/DS do not contain the absorbance band of an unsymmetrical MB dimer at 718 nm that was observed for MB adsorbed on the interlayer surface of hectorite [43]. The existence of the MB dimers on the external surface is confirmed by the adsorption band at 615 nm. The absorption at 673 nm that is typical for the MB monomers adsorbed on the external surface [43] was absent. Thus, the interlayer space of LDHs included monomers of MB and the external LDHs surface contained MB dimmer forms. Evidently, the organo/Zn-Al LDHs consist of DS in two forms: intercalated into the interlayer galleries of the LDHs and adsorbed on the surface.

The diffuse reflectance spectra of ZnAl LDH/DS with higher loading of MB (110 mg g^−1^) show the broadening of absorption to 450 nm (Fig. 7, #3). The absorption of MB trimer state is observed at 570 nm [42, 43]. It is possible that aggregates with larger than three units are formed on the surface of ZnAl LDH/DS with higher MB loading.

The adsorption capacity of ZnAl LDH/DS was compared with the other sorbents (Table 5). These selected data suggest that organo/Zn-Al LDHs are promising materials with complementary properties to activated carbon and clay materials.

**Table 5:** Adsorption capacity of (*q*_max_ calculated from Langmuir model) of adsorbed MB by several adsorbents

Adsorbents	Adsorption capacities, mg g^−1^	Sources
Activated carbon (commercial)	10	[45]
Bituminous coal (commercial)	176	[46]
Filtrasorb 400	299	[47]
Spent activated clay	128	[48]
Palygorskite	51	[49]
Montmorillonite clay	289	[50]
Mg–Al LDH	49	[51]
MgAl/DS LDH-iron oxide	110	[22]
ZnAl/DS LDH	113	This study

**Fig. 8:**
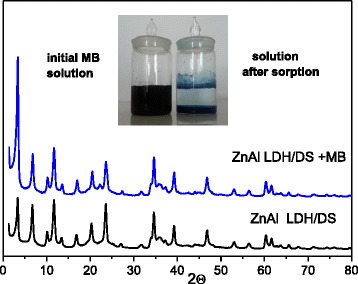
XRD patterns of ZnAl LDH/DS before and after MB sorption

To check the resistance of organo/LDHs relatively leaching of DS ions, the structure of ZnAl LDH/DS after sorption of MB was analyzed. No shift in the diffraction peak for the 2*θ* value equal to 3.4° was observed at XRD pattern of ZnAl LDH/DS with sorbed MB (*q* = 32 mg g^−1^) (Fig. 8). Evidently, the configuration of intercalated DS is not visibly affected by MB sorption process. DS is rigidly fixed in the interlayers of ZnAl LDH/DS; consequently, the interlayer spacing remains unchanged even after the introduction of the second organic molecules. Thus, the stability of organo/LDHs has been found to be high under stirring in MB aqueous solutions. The increase of the integral intensity of diffraction peak related to the intercalated phase was observed for ZnAl LDH/DS with sorbed MB. The integral intensities ratio of reflections that corresponded to intercalated (2*θ* = 3.4°) and hydrotalcite (2*θ* = 11.7) phases was 1.9. This is more than two times higher that integral intensities ratio for ZnAl LDH/DS before MB sorption (Table 2). Apparently, the additional formation of intercalated crystalline phase occurs in ZnAl LDH/DS during MB sorption. Probably, MB molecules initiate the transformation of the amorphous phase of DS-intercalated ZnAl LDHs into the crystalline phase. It should be noted that the increase of the amount of intercalated phase was observed only for ZnAl LDH/DS stirred in MB solutions. The integral intensities ratio and peak position in XRD patterns of ZnAl LDH/DS were unchanged after stirring in an aqueous suspension.

## Conclusions

The modification of ZnAl LDHs in DS solutions by reconstruction method is the facile way of preparation of organo/LDH nanohybrids for the sorption of cationic dye MB from the aqueous medium. The reconstructed organo/Zn-Al LDHs contained as the crystalline (DS-intercalated LDH and hydrotalcite structure), so amorphous phases. The internal and external surfaces of LDHs are modified with DS that causes MB molecules embedding in the interlayers and its adsorption on the surfaces of LDHs. The organo/LDH sorbents are stable to leaching of DS anions from the interlayer spacing. The study of pH effect on MB sorption by ZnAl LDH/DS leads to the conclusion that the hydrophobic/hydrophobic interactions between the alkyl chains of the surfactant and MB molecules are predominated over the electrostatic attractions. Despite the fact that the organo/ZnAl LDHs contained the DS-intercalated phase and hydrotalcite phase, they showed sorption capacity to MB, comparable to LDHs composites, which exclusively consisted of DS-intercalated phase. The colored organo/Zn-Al LDHs are easy to separate from the solution, and the resulting sludge could be reutilized in other applications like as pigments, colorant additives for polymers, and light-activated antimicrobial surfaces.

The current research work provides a further insight into the applications of LDHs-based composites as surfactants and dyes sorbents for wastewater treatment.
